# bwtool: a tool for bigWig files

**DOI:** 10.1093/bioinformatics/btu056

**Published:** 2014-01-30

**Authors:** Andy Pohl, Miguel Beato

**Affiliations:** ^1^Department of Gene Regulation, Stem Cells, and Cancer, Centre for Genomic Regulation (CRG) and ^2^Department of Experimental and Health Sciences (CEXS), Universitat Pompeu Fabra, 08003 Barcelona, Spain

## Abstract

BigWig files are a compressed, indexed, binary format for genome-wide signal data for calculations (e.g. GC percent) or experiments (e.g. ChIP-seq/RNA-seq read depth). bwtool is a tool designed to read bigWig files rapidly and efficiently, providing functionality for extracting data and summarizing it in several ways, globally or at specific regions. Additionally, the tool enables the conversion of the positions of signal data from one genome assembly to another, also known as ‘lifting’. We believe bwtool can be useful for the analyst frequently working with bigWig data, which is becoming a standard format to represent functional signals along genomes. The article includes supplementary examples of running the software.

**Availability and implementation:** The C source code is freely available under the GNU public license v3 at http://cromatina.crg.eu/bwtool.

**Contact:** andrew.pohl@crg.eu, andypohl@gmail.com

**Supplementary information:** Supplementary data are available at *Bioinformatics* online.

## 1 INTRODUCTION

For many laboratories, it has become an everyday task to generate or to analyze genome-wide data such as ChIP-seq read depth. To facilitate visualization of these data with tools such as the UCSC Genome Browser (Kent *et al.*, 2002) or ENSEMBL (Flicek *et al.*, 2013), or for further processing, it is common to use the wiggle (WIG) file format. This format is not without a few disadvantages, principally that the files can become large, particularly when care is not taken to store the data at a minimally necessary decimal precision. Another disadvantage is that wiggles exist in three different forms, the choice of which depends on the sparseness of the data. Programs that expect WIG data do not always allow all three formats interchangeably.

The bigWig format (Kent *et al.*, 2010) was created as a means for the UCSC Genome Browser to access real-valued signal data remotely hosted on HTTP/FTP servers worldwide. The format is binary, compressed, indexed and allows random access to directly query a subset of the larger dataset. In general, programs designed to read bigWig files should treat remote URLs of bigWigs the same as they would treat URLs local to that computer. BigWig uses an indexing strategy similar to other binary/indexed formats such as bigBed (Kent *et al.*, 2010), binary SAM (BAM) (Li *et al.*, 2009) and tabix-based formats (Li, 2011), but unlike BAM or tabix-based formats, bigWig is specific to numerical data. WIG and BAM are both common data formats and are used by many applications, e.g. Model-based analysis of ChIP-Seq (MACS) (Zhang *et al.*, 2008) and Mixture of Isoforms (MISO) (Katz *et al.*, 2010), respectively, but, to date, there are not many applications that accommodate bigWig data.

We have created command-line software under the UNIX operating system called bwtool in a similar spirit to bedtools (Quinlan and Hall, 2010) or samtools (Li *et al.*, 2009) that offers the possibility to carry out a number of diverse operations on bigWigs in a convenient way. Until now, the common procedure to access the data within bigWig files has been to use the tools available from UCSC: bigWigToWig, bigWigSummary, bigWigAverageOverBed, bigWigMerge, bigWigCorrelate or bigWigInfo. These offer some basic usability for bigWigs. bigWigInfo provides instant information about a bigWig file and is useful for glancing at the overall mean and standard deviation as well as seeing how many bases are covered by the signal. bigWigToWig is indispensible, as it is occasionally necessary to convert a bigWig into the original WIG to use legacy software. Beyond those two, bwtool provides additional features and flexibility not found in other software.

## 2 DESCRIPTION

**Fig. 1.**
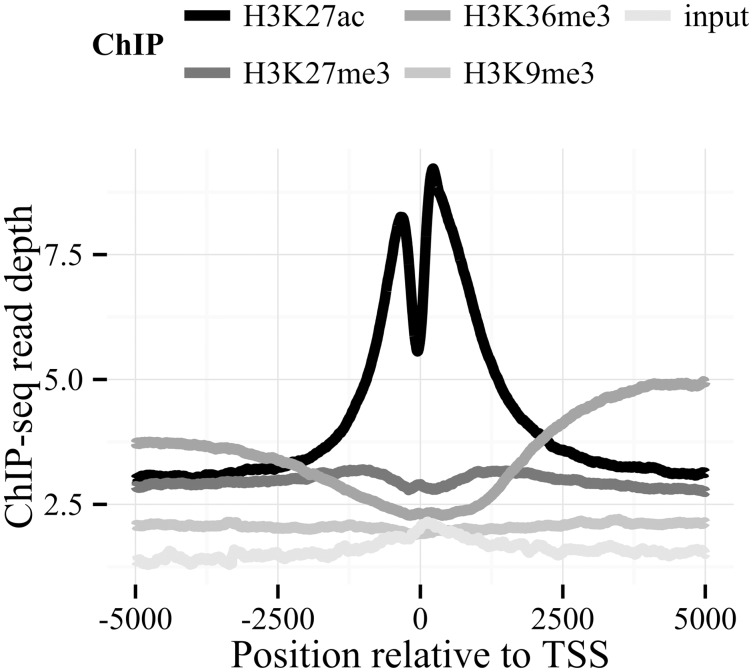
Example of aggregated plots of different histone modification ChIP sequence read-depth signals from MCF7 cells from ENCODE aligned at each of the 20 330 protein-coding gene transcription start sites in GENCODE release v17 (Harrow *et al.*, 2012). See Supplement for instructions on how to reproduce this plot. The raw signals in this example are not normalized, so specific values cannot be compared between signals; however, the morphological differences in averaged profiles are nevertheless useful in characterizing the patterns of each histone mark

The bwtool program is designed to rapidly collect summary statistics and do common wiggle manipulations. The program is a collection of utilities (the names of which are in bold), which allow for the following features:

- **Aggregate** data by averaging it over a series of given intervals with respect to central bases. This common aggregation procedure is used to produce plots showing enrichment, but has a tendency to be problematic, particularly when centering on genomic features without a known strand or directionality (Kundaje *et al.*, 2012). For this reason, simple k-means functionality is built-in to group regions with similar profiles. Figure 1 demonstrates the aggregate program on data collected from the ENCODE project (Raney *et al.*, 2011).
- ‘**Lift**’, i.e. project data from one genome assembly to another using a ‘liftOver chain’ file, available from the UCSC Genome Browser utilities page (Kuhn *et al.*, 2013). Lifting data often results in a small percentage of data loss, so care must be taken to ensure that the only lifted data analyzed is that which is within regions lifting correctly. Options are available to catalog all of the problematic regions involved.
- Quickly **find** regions in the bigWig exhibiting local minima/maxima, or above/below specified thresholds.
- Extract equally sized intervals of data as a **matrix** or as a sliding **window** at adjustable steps and sizes. Again, clustering is available as an option when extracting data as a matrix. A **random** matrix of data can also be produced, with the ability to exclude specific regions in the genome. Unequally sized intervals can be also extracted with the **extract** utility.
- Another way to extract data from multiple bigWigs is to use the **paste** utility. This outputs tab-delimited data from a set of bigWigs, one base per line. Pasting bigWigs together makes it possible to perform many complex calculations with small auxiliary scripts. In this way, the functionality of bwtool can easily expand on the functionality of bigWigMerge and bigWigCorrelate from UCSC.
- Discretize the real-valued signal into letters, using the Symbolic Aggregate Approximation (SAX) algorithm (Shieh and Keogh, 2009).
- **Removing** data based on thresholds and specific regions if desired. Conversely, regions missing data in a bigWig can be replaced with a constant using the **fill** utility.
- **Summarize** data at specific regions. This functionality is similar to the combined programs of bigWigSummary and bigWigAverageOverBed, with the addition of median and optional quantile information in the output.

Common options to many of the features include the ability to specify the decimal precision, to fill missing bases with a given value or to provide a bed file specifying specific regions of the bigWig to read.

## 3 USAGE AND AVAILABILITY

bwtool is command-line software for UNIX, a common platform for bioinformatics researchers to conduct analysis. Running the bwtool command without additional parameters displays a description of the various utilities and some general options. Combined with a utility name, bwtool will display specific information about how to perform an operation using that utility. A detailed guide has been created on bwtool's web page (http://cromatina.crg.eu/bwtool) to provide thorough examples of using the program.

bwtool is written in C. The source code for the program is available on its GitHub web page. Distributed (with permission) with bwtool is the basic C library from Jim Kent that is needed for routines specific to bigWig data, as well as other algorithmic code. Jim Kent and the University of California hold the copyright to this specific library, but the remaining code is covered by the GNU Public License v3. bwtools makes use of GNU autotools to simplify the installation process to the standard ‘./configure’, ‘make’, ‘make install’ procedure most UNIX users will be familiar with. To verify the accuracy of the software, tests may be run with ‘make check’. bwtool does not require additional libraries that are not typically found in common UNIX environments, but if the GNU Scientific Library is installed, it will make use of that for the random utility.

## Supplementary Material

Supplementary Data
