# Supplementary material for: bwtool: a tool for bigWig files
Source: Bioinformatics. 2014 Jan 30;30(11):1618–9. doi: 10.1093/bioinformatics/btu056 (PMC4029031; doi:10.1093/bioinformatics/btu056)
Supplement: Supplementary Data [file supp_30_11_1618__index.html]

bwtool: A tool for bigWig files — bwtool: a tool for bigWig files — bwtool: a tool for bigWig files — Supplementary Data 

# bwtool: a tool for bigWig files

## Supplementary Data

files

**Files in this Data Supplement:**

- Supplementary Data - pdf file
