# Supplementary material for: bwtool: a tool for bigWig files
Source: Bioinformatics. 2014 Jan 30;30(11):1618–9. doi: 10.1093/bioinformatics/btu056 (PMC4029031; doi:10.1093/bioinformatics/btu056)
Supplement: Supplementary Data [file supp_btu056_Supplement.pdf]

Supplement

In order to create Figure 1 from the main text, R and ggplot2 are needed. If these are installed, follow these steps:

1. Download and install bwtool:

```
$  git clone https://github.com/andypohl/bwtool.git
$  cd bwtool
$  ./configure   (or  ./configure –prefix=$HOME  if root access is unavailable)
$  make
$  make check
$  make install
```

2. [*]Download data (available as of 8th October 2013) from GENCODE and UCSC:

```
$  curl -O ftp://ftp.sanger.ac.uk/pub/gencode/release_17/gencode.v17.annotation.gtf.gz
$  curl http://hgdownload.cse.ucsc.edu/goldenPath/hg19/encodeDCC/wgEncodeSydhHistone/wgEncodeSydhHistoneMcf7H3k27acUcdSig.bigWig -o H3K27ac.bw
$  curl http://hgdownload.cse.ucsc.edu/goldenPath/hg19/encodeDCC/wgEncodeSydhHistone/wgEncodeSydhHistoneMcf7H3k09me3UcdSig.bigWig -o H3K9me3.bw
$  curl http://hgdownload.cse.ucsc.edu/goldenPath/hg19/encodeDCC/wgEncodeSydhHistone/wgEncodeSydhHistoneMcf7H3k27me3bUcdSig.bigWig -o H3K27me3.bw
$  curl http://hgdownload.cse.ucsc.edu/goldenPath/hg19/encodeDCC/wgEncodeSydhHistone/wgEncodeSydhHistoneMcf7H3k36me3bUcdSig.bigWig -o H3K36me3.bw
$  curl http://hgdownload.cse.ucsc.edu/goldenPath/hg19/encodeDCC/wgEncodeSydhHistone/wgEncodeSydhHistoneMcf7InputUcdSig.bigWig -o input.bw
```

3. Create a file from the GENCODE annotation containing merely the basic coordinates of the protein-coding genes i.e. chromosome, TSS, TTS, name, and strand. Proper bed files have a score as the fifth field and strand as the sixth field. Scores are not used by bwtool so can be set to zero:

```
$ zcat gencode.v17.annotation.gtf.gz \
   | awk 'BEGIN{OFS="\t"}{if($3=="gene" && $20=="\"protein_coding\";"){print $1, $4-1, $5, $18, "0", $7}}' \
   | sed 's/\"//g;s/;//' | sort -k1,1 -k2,2n > gencode_pc.bed
```

4. Run bwtool:

```
$ bwtool agg 5000:5000 -starts -long-form=TSS,H3K27ac,H3K27me3,H3K36me3,H3K9me3,input gencode_pc.bed \
H3K27ac.bw,H3K27me3.bw,H3K36me3.bw,H3K9me3.bw,input.bw plot.txt
```

5. From inside R:

```
>  library(ggplot2)
>  library(extrafont)
>  plots <- read.table('plot.txt')
>  colnames(plots) <- c('Feature','ChIP','Position','Signal')
>  figure<-ggplot(plots, aes(x=Position,y=Signal,color=ChIP)) + geom_line(size=1.5) + theme_minimal() + scale_color_grey(start = 0, end = .9) +
theme(text=element_text(family="Times New Roman"), legend.position="top") + guides(col=guide_legend(nrow=2)) + ylab("ChIP Read Depth") +
xlab("Position relative to TSS")
>  figure
>  ggsave("figure.png", height=3.4, width=3.4, units="in", dpi=1200)
```

More examples, including smaller ones, can be found on the web page: http://cromatina.crg.eu/bwtool

[*] For faster downloads, use instructions on the next page for download step.

If a fast internet connection is unavailable, there are several options to aid this issue:

1. Use the **paraFetch** utility in place of **curl** or **wget** *(recommended)*. paraFetch opens multiple HTTP connections simultaneously and in practice tends to download large files several times faster. paraFetch is available from http://hgdownload.cse.ucsc.edu/admin/exe/. For example:

```
$ time curl http://hgdownload.cse.ucsc.edu/goldenPath/hg19/encodeDCC/wgEncodeSydhHistone/wgEncodeSydhHistoneMcf7H3k27acUcdSig.bigWig -o H3K27ac.bw
  % Total    % Received % Xferd  Average Speed   Time    Time     Time  Current
                                 Dload  Upload   Total   Spent    Left  Speed
100  256M  100  256M    0     0   936k      0  0:04:40  0:04:40 --:--:--  959k

real 4m41.438s
user 0m0.350s
sys  0m1.059s
```

is slower than:

```
$ time paraFetch 20 10 http://hgdownload.cse.ucsc.edu/goldenPath/hg19/encodeDCC/wgEncodeSydhHistone/wgEncodeSydhHistoneMcf7H3k27acUcdSig.bigWig
H3K27ac.bw

real  0m29.828s
user  0m0.082s
sys   0m2.555s
```

2. Download only the H3K27ac and input bigWigs. In this case, the resulting bwtool command will look like:

```
$ bwtool agg 5000:5000 -starts -long-form=TSS,H3K27ac,input gencode_pc.bed H3K27ac.bw,input.bw plot.txt
```

3. Use reduced versions of the original bigWigs, listed here:

```
http://cromatina.crg.eu/bwtool/figure/MCF7H3K27ac.tss-10kb.bw
http://cromatina.crg.eu/bwtool/figure/MCF7H3K27me3.tss-10kb.bw
http://cromatina.crg.eu/bwtool/figure/MCF7H3K36me3.tss-10kb.bw
http://cromatina.crg.eu/bwtool/figure/MCF7H3K9me3.tss-10kb.bw
http://cromatina.crg.eu/bwtool/figure/MCF7input.tss-10kb.bw
```

In this case, the bigWigs were reduced from genome-wide datasets to only the bases +/- 5000 bases from transcription start sites. **bwtool remove** was used in this case, in the following way:

```
$ curl –O http://cromatina.crg.eu/bwtool/hg19.sizes
$ cat gencode_pc.bed | awk 'BEGIN{OFS="\t"}{if ($6 == "+") { $3 = $2 + 5001; $2 = $2 - 5001;} else { $2 = $3 - 5001; $3 = $3 + 5001;} print;}' |
sort -k1,1 -k2,2n | bedClip /dev/stdin hg19.sizes /dev/stdout | bedtools merge > tss.bed
$ for f in MCF7*.bw; do  bwtool remove mask tss.bed –inverse –decimals=0 $f ${f%.bw}.tss-10kb.bw; done
```

(needs **bedtools** and the **bedClip** utility from http://hgdownload.cse.ucsc.edu/admin/exe/). In this case, the relevance of the reduced bigWigs may be verified by loading the bigWigs as tracks in the UCSC Genome Browser using the "track hub" feature with the following custom track hub link: http://cromatina.crg.eu/bwtool/hub.txt.
